# Weight estimation and hormone concentrations related to body condition in Icelandic and Warmblood horses: a field study

**DOI:** 10.1186/s13028-019-0498-5

**Published:** 2019-12-26

**Authors:** Rasmus Bovbjerg Jensen, Lucca Louise Rockhold, Anne-Helene Tauson

**Affiliations:** 1 Department of Veterinary and Animal Sciences, Faculty of Health and Medical Sciences, University of Copenhagen, 1870 Frederiksberg C, Denmark; 2 Department of Animal and Aquacultural Sciences, Faculty of Bio Sciences, Norwegian University of Life Sciences, 1433 Ås, Norway

**Keywords:** Body weight, Cresty neck score, Leptin, Morphological measurements, Obesity

## Abstract

**Background:**

The main objectives of this study were to evaluate the accuracy of different body weight formulas for estimating body weight of Icelandic and Warmblood horses, as well as to assess the associations between the variables cresty neck score, body condition score, and plasma concentrations of leptin, insulin and cortisol. A total of 81 adult (≥ 4 years of age) horses (43 Icelandic and 38 Warmblood horses) was included in this study. The following morphological measurements were collected by two examiners simultaneously; body weight; height at withers; neck length; 0.5 neck length; neck circumference; umbilical circumference, two different heart girths, as well as two different body length measurements. The horse’s body weights were measured on a weight scale, and cresty neck scores were rated along with body condition scores using both the 0 to 5 and the 1 to 9 systems.

**Results:**

In general, the concordance correlation coefficient was high for most formulas, but the mean bias and slope bias deviations varied between formulas. Some simple formulas using only heart girth, or heart girth and length can be used to estimate body weight of Icelandic and Warmblood horses as good as more complex formulas using four morphometric measurements. Plasma concentrations of leptin and insulin were higher (P < 0.001) for the Icelandic than the Warmblood horses, probably reflecting higher body fat content as suggested by the differences in body condition score.

**Conclusions:**

Body weight formulas only give an estimate of body weight and not a completely correct determination. Some simple and more complex formulas can be used for Icelandic horses even though they are not developed for this breed. Complex formulas using four morphometric measurements were accurate, but simple formulas using only heart girth, or heart girth and length can be used to estimate body weight and thereby be applied to weight tapes and used to estimate the body weight of both Icelandic and Warmblood horses.

## Background

The ability to precisely and accurately obtain and monitor horses’ body weight (BW), as well as body condition score (BCS) and cresty neck score (CNS) is essential for generating an optimal nutritional plan and monitoring horse health. Further, with equine obesity being a major health issue on the rise, the ability to recognize a horse as overweight or obese and monitor BW fluctuations on a regular basis is becoming increasingly important [1–4].

A weight scale is the most accurate way of obtaining the BW of a horse, but it is often not accessible. Without availability of a weight scale, a horse’s BW is commonly visually estimated, but this is an inaccurate way of assessing BW [5, 6], and it has been found that the mean accuracy was 98.6 vs. 88.3% when estimating the BW with a formula or by visual assessment, respectively [5]. Body weight is more appropriately estimated by using BW estimation methods such as weight tapes and BW formulas. These methods use morphometric measurements to acquire an estimated BW. The weight tape uses only the girth circumference to determine the horse’s BW because girth circumference is highly correlated with BW [7]. A BW formula (BWF) can be more complex, but has also been suggested to be more accurate than a weight tape, and several BWF have been developed over the years [5, 8]. However, the conformational differences between various breeds may influence the accuracy of the respective BWF. Therefore, it is important to evaluate different BWFs and their suitability for use in different breeds, e.g. only one study has evaluated the suitability of a BWF [7] in a limited number (n = 13) of Icelandic horses [9]. Hence, more research is needed to clarify if BWF developed for other breeds are suited for Icelandic horses.

The main objective of this study was to evaluate the accuracy of different BW formulas for estimating BW of two different horse breeds, the Icelandic and Warmblood horse, as well as to assess the associations between the variables CNS, BCS, plasma concentrations of leptin, insulin and cortisol.

## Methods

### Experimental design and animals

The experiment was a field study conducted in Denmark and included horses located at six different stables. A total of 81 horses was assessed during this study, involving 43 Icelandic and 38 Warmblood horses. There was a total of 52 geldings (20 Icelandic and 32 Warmblood horses with an average age of 12 ± 5.7 and 11 ± 3.3 years, respectively), 4 stallions (4 Icelandic horses with an average age of 10 ± 2.6) and 25 mares (19 Icelandic and 6 Warmblood horses with an average age of 9 ± 3.3 and 15 ± 4.3 years, respectively). The age of 12 Icelandic geldings and 7 Icelandic mares were not registered, but horses that were < 4 years of age, as well as pregnant or lactating mares were excluded from the study. The horses were not fasted or prohibited from water intake before being examined. All horses were in good health and on a vaccination, dental care and deworming management plan, according to the owners.

### Morphometric measurements

**Fig. 1:**
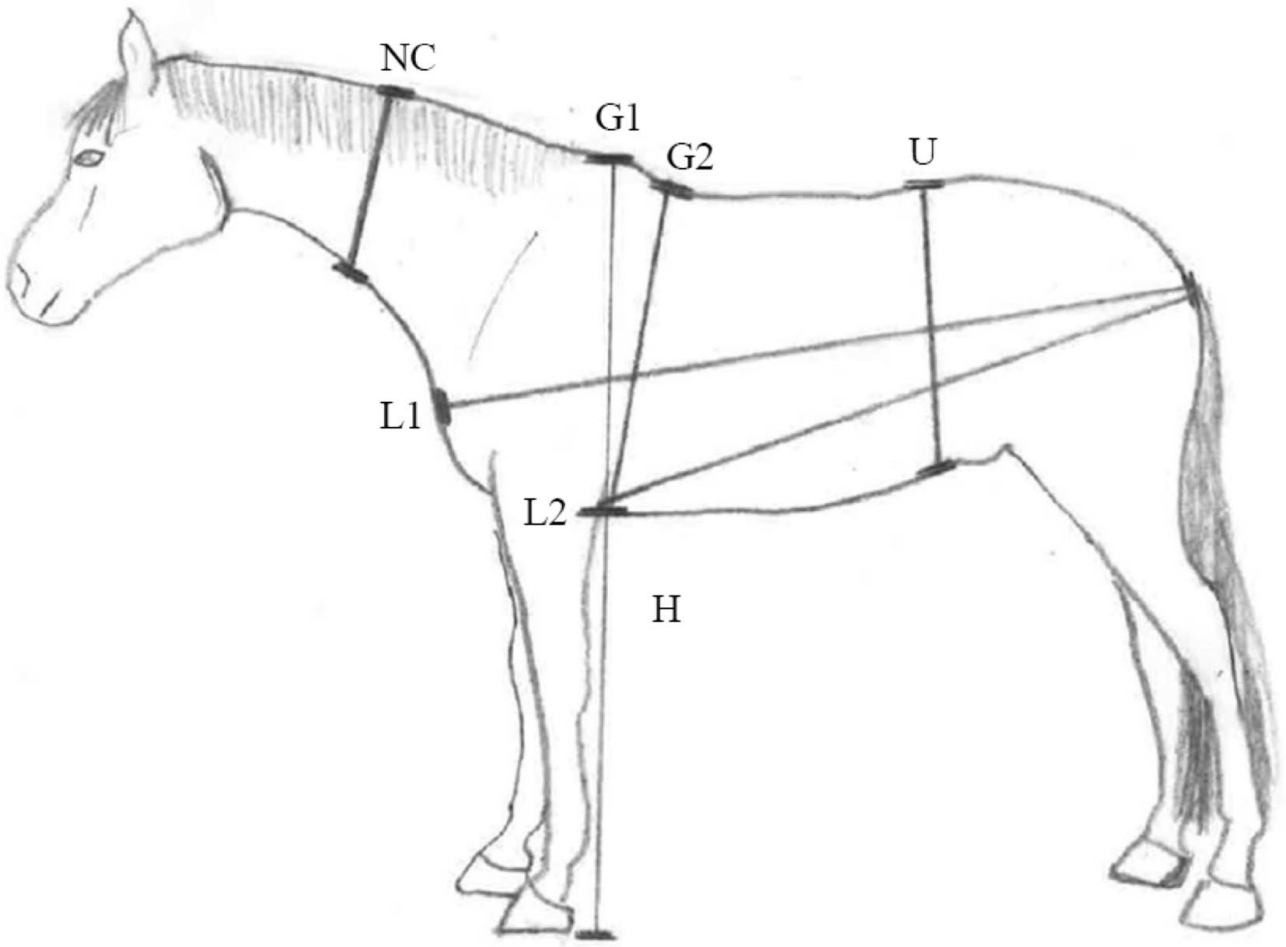
A depiction of all the morphometric measurements. NC: neck circumference; L1: length from point of shoulder to point of buttock; L2: length from point of elbow to point of buttock; G1: heart girth from top of the withers; G2: heart girth from slope of the withers; U: umbilical girth; H: height at withers. All morphometric measurements were obtained in centimetres

Each horse’s body weight, measured on a weight scale, and morphometric measurements (Fig. 1) were taken at the same time to avoid any fluctuations in BW. To ensure consistency the same two examiners preformed all of the morphometric measurements simultaneously and measurements were taken once.

Each horse was first weighed using a portable weight scale (Horse weigh® “Tokyo” EziWeigh; Tru-Test, Wales, UK), with an accuracy of ± 1% according to the manufacturer. The horse’s BW was rounded to the nearest kg. To ensure accurate readings the weight scale was placed on a hard and flat surface and the scale was tested with an object of known weight. After being weighed, the horse was situated on level ground in a square stance. Thereafter, morphometric measurements were taken, starting with height at withers (H). Height at withers was taken using a measuring stick that was placed at the base of the horse’s hooves and measured up to the highest point of the withers. In a relaxed and natural neck position, the neck length (NL) and neck circumference (NC) were measured. The NL was taken from the poll to the highest point of the withers following the curvature of the neck and the NL was only used to locate where to measure NC. The NC was taken from the halfway point of the NL, wrapping around the whole neck. Heart girth circumference was taken at two locations, both starting from behind the elbow and wrapping vertically around the sternum of the horse to the highest point of the withers (G1) and the other to the base (slope) of the withers (G2). The umbilical girth circumference (U) was taken from the umbilicus point going vertically around the abdomen. All girth circumferences were obtained at the time of respiratory expiration. This was done to avoid girth circumference increase due to inhalation. Two body lengths were taken, one in a horizontal line from the tuber ischium (point of the buttocks) to greater tubercle (point of the shoulder) (L1) and the other from the tuber ischium (point of the buttocks) to olecranon (point of the elbow) (L2). Figure 1 depicts all of the morphometric measurements.

### Formulas

The morphometric measurements gathered were used to obtain indirect measurements of the horses BW using the formulas presented in Table 1.

**Table 1:** The different body measurements of horses and formulas used to estimate body weight (BW) of horses

Reference	Measurements^c^	BW formula (kg)
Marcenac and Aublet [10]	G2	Girth (m)^3^ × 80
Staun [11]	G2	6.25 × Girth (cm) – 625
Willoughby [12]^a^	G2	(0.14475 × Girth (cm) / 2.54)^3^ × 0.4536 (Males) (0.14341 × Girth (cm) / 2.54)^3^ × 0.4536 (Females)
Ensminger [13]	G1 + L1	((Girth^2^ (cm) × Length (cm))/2.54^3^/660 + 22.7
Jansson [14]	G2 + L2	Girth^2^ (cm) × Length (cm)/8900
Carroll and Huntington [7]^b^	G2 + L1	(Girth^2^ (cm) × Length (cm))/11,877
Jones et al. [15]	U + L1	(Girth (cm)^1.78^ × Length (cm)^0.97^)/3011
Martin-Rosset [16]	H + G2	4.3 × Girth (cm) + 3.0 × Height (cm) – 785 (Horse) 3.56 × Girth + 3.65 × Height – 714.77 (Pony)
Martinson [17]	H + NC + G1 + L1	(Girth (cm)^1.486^ × Length (cm)^0.554^ × Height (cm)^0.599^ × Neck (cm)^0.173^)/3606
Catalano [18]	H + NC + G1 + L1	(Girth (cm)^1.528^ × Length (cm)^0.574^ × Height (cm)^0.246^ × Neck (cm)^0.261^)/1209

G1: heart girth from top of the withers; G2: heart girth from slope of the withers; L1: length from point of shoulder to point of buttock; L2: length from point of elbow to point of buttock; U: umbilical girth, H: height at withers; NC: neck circumference

^a^In the original reference units are in inches (in) and pounds (lb)

^b^If BCS < 2.5 out of 5 use Y derivative 12265, if BCS ≥ 3 out of 5 then use Y derivative 11706

^c^Measurements are illustrated in Fig. 1

### Cresty neck and body condition score

The horses’ BCSs were evaluated using the 0 to 5 BCS [7] and 1 to 9 BCS [19] systems. The Carroll and Huntington [7] BCS, ranges from 0 (horse is emaciated) to 5 (horse is extremely fat), where a horse given a score of 2 or 3 is considered to be in a moderate respectively good body condition. Henneke et al. [19] BCS ranges from 1 (emaciated) to 9 (extremely fat). For the 1 to 5 BCS, the pelvic score was adjusted by 0.5 points if it differed by 1 or more points from the back and neck score as described by Carroll and Huntington [7]. The CNS was evaluated using the CNS developed by Carter et al. [20]. The CNS scores range from 0 (no crest) to 5 (large crest that drops to one side), with a score of ≥ 3 indicating a CN [20]. Half point increments are applied to both the CNS and 1 to 9 BCS. This was done to allow for better allocation of horses that are intermediate to the specifications of two whole BCS numbers.

### Blood samples and hormone analysis

Blood samples were collected by jugular vein puncture into 10 mL heparinized tubes (BD vacutainer sodium heparin, Becton, Dickinson and Company, Franklin Lakes, NJ, USA) from 73 of the 81 horses (35 Icelandic and 38 Warmblood horses) before feeding (blood samples and morphometric measurements were performed on different days). The blood samples were centrifuged (Hettich centrifuge EBA 200, Andreas Hettich GmbH & Co.KG, Tuttlingen, Germany) immediately after sampling at 3000×*g* for 10 min, plasma was harvested, placed on ice and stored at − 20 ℃ within 1 h for later analysis of plasma hormones.

Plasma was analysed for leptin, insulin and cortisol as described in Jensen et al. [21]. The samples were processed in a single assay with a detection limit of 0.04 ng/mL, 1.4 µU/mL and 3.56 ng/mL for leptin, insulin and cortisol, respectively. For leptin, control samples containing 0.06, 0.27 and 1.03 ng/mL were included in the assay and used to estimate intra-assay coefficients of variation of 10.5, 4.5 and 4.6%, respectively. For insulin, control samples containing 3.98, 7.82 and 16.36 µU/mL were included in the assay and used to estimate intra-assay coefficients of variation of 4.2, 8.1 and 1.6%, respectively. For cortisol, control samples containing 11.07 and 22.51 ng/mL were included in the assay and used to estimate intra-assay coefficients of variation of 6.3 and 4.0%, respectively.

### Calculations and statistical analyses

The effects of breed on morphometric measurements, CNS, BCS and BW were analysed using PROC MIXED in SAS® (Version 9.4, SAS Institute Inc., Cary, NC, USA) in a model where breed was included as a fixed effect. The Bland–Altman plot [22] was used to assess the accuracy of the BWF both visually and statistically by comparing measured BW and estimated BW in GraphPad Prism® (Version 7, GraphPad Software, La Jolla, California, USA). The y-axis in the Bland–Altman plot is the difference between the measured and estimated BW (mean ± SD) and the x-axis is the average of the measured and estimated BW. The grey area represents the 95% confidence interval. Ideally, all data points should be evenly distributed, lie within the 95% confidence interval and the mean should be close to zero.

A combination of formula evaluation metrics was also used to assess formula performance including root mean square prediction error (RMSPE), RMSPE to standard deviation of the measured BW ratio (RSR), mean bias (MB) and slope bias (SB) deviations, and concordance correlation coefficient (CCC) according to Niu et al. [23] and analysed using R® statistical software.

The RMSPE was used to assess overall formula prediction accuracy and its units are the same as the measurements:

$$\text{R}\text{M}\text{S}\text{P}\text{E} =\frac{{{\sum }_{i=1}^{n}\left({Y}_{i}-{ \hat{Y} }_{i}\right)}^{2}}{n}$$

where *Y*_*i*_ denotes the measured BW for the *i*th observation, *Ŷ*_*i*_ denotes the predicted BW for the *i*th observation, and *n* denotes the number of observations. The RSR was caluculated as:

$$\mathrm{R}\mathrm{S}\mathrm{R}=\frac{RMSPE}{{S}_{O}}$$

where *S*_*o*_ denotes the standard deviation of the measurements. Smaller RSR indicates better model predictive ability given the variability of the data. To identify systematic biases, the MB and SB deviations were calculated:

$$MB={(\stackrel{-}{P}-\stackrel{-}{O})}^{2}$$

$$SB={({S}_{p}-r \times { S}_{o})}^{2}$$

where $\stackrel{-}{P}$ and $\stackrel{-}{O}$ denotes the predicted and the measured means, ${S}_{p}$ denotes the standard deviation of the predicted values, and *r* denotes the Pearson correlation coefficient. To evaluate the degree of deviation between the best-fit line and the identity line (*y* = *x*) the CCC was calculated, and a value closer to 1 indicates a better model performance:

$$CCC={r \times { C}_{b}}^{2}$$

where

$${C}_{b}={\left[\left(\left(v+1\right)/\left(v+{u}^{2}\right)\right)/2\right]}^{-1}$$

$$v= {S}_{o}/{S}_{p}$$

$$u={(\stackrel{-}{P}-\stackrel{-}{O})/ ({S}_{o}{S}_{P})}^{1/2}$$

where $\stackrel{-}{P}$, $\stackrel{-}{O}$, ${S}_{o}$, and *S*_*o*_ are defined above, *v* provides a measure of scale of shift, and *u* provides a measure of location shift.

The effects of breed and BCS on hormone concentrations were analysed using PROC MIXED in SAS® using a model where breed and BCS were included as fixed effect (BCS was not significant and removed from the model), and associations between the variables CNS, BCS (1–5), BCS (1–9), leptin, insulin and cortisol were tested with Spearman’s rank correlation coefficient (r_s_) using procedure REG in SAS®. Results are presented as least square means (LS-means) with standard error of the mean (SEM) as a measure of variance. Effects were considered significant if P < 0.05.

## Results

The morphometric measurements differed (P < 0.01) between the two horse breeds and LS-mean as well as minimum and maximum values of H, G1, G2, U, L1, L2, NC, CNS, BCS (0–5), BCS (1–9) and BW are presented in Table 2.

**Table 2:** Morphometric measurements, cresty neck score (CNS), body condition score (BCS) and body weight (BW) of Icelandic and Warmblood horses

	Icelandic (n = 43)				Warmblood (n = 38)				P-value
	Mean	SEM	Min	Max	Mean	SEM	Min	Max	
H (cm)	138	0.63	130	143	172	0.67	158	180	< 0.001
G1 (cm)	166	0.86	151	180	204	0.91	189	213	< 0.001
G2 (cm)	164	0.86	150	176	199	0.92	184	212	< 0.001
U (cm)	170	1.2	150	192	204	1.3	178	216	< 0.001
L1 (cm)	158	1.0	146	176	185	1.1	169	196	< 0.001
L2 (cm)	128	0.84	116	140	153	0.89	139	163	< 0.001
NC (cm)	87	0.88	77	104	98	0.94	88	110	< 0.001
CNS	2.1	0.07	1	3.5	1.4	0.09	0.5	2.5	< 0.001
BCS (0–5)	3.0	0.05	2.2	3.5	2.8	0.05	1.5	3	< 0.001
BCS (1–9)	5.7	0.09	4.5	7	5.3	0.10	4	6	< 0.01
Measured BW	366	6.4	294	428	640	6.8	478	718	< 0.001

H: height at withers; G1: heart girth at withers; G2: heart girth at slope; U: umbilical girth; L1: length from point of shoulder to point of hip; L2: length from point of elbow to point of hip; NC: neck circumference; CNS: cresty neck score; BCS (0–5): body condition score on scale 0–5; BCS (1–9): body condition score on scale 1–9; BW: body weight; SEM: standard error of the mean; Max: maximum; Min: minimum.

**Fig. 2:**
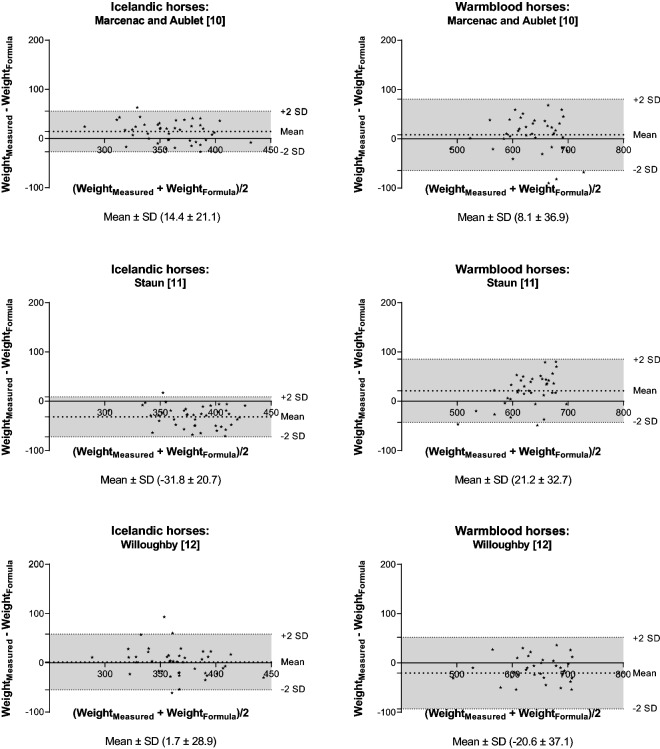
Bland–Altman plot of the measured and estimated body weight (BW). The BW of Icelandic and Warmblood horses was estimated only based on heart girth. The y-axis is the difference between the measured and estimated BW (mean ± SD) and the x-axis is the average of the measured and estimated BW. The grey area represents the 95% confidence interval

**Fig. 3:**
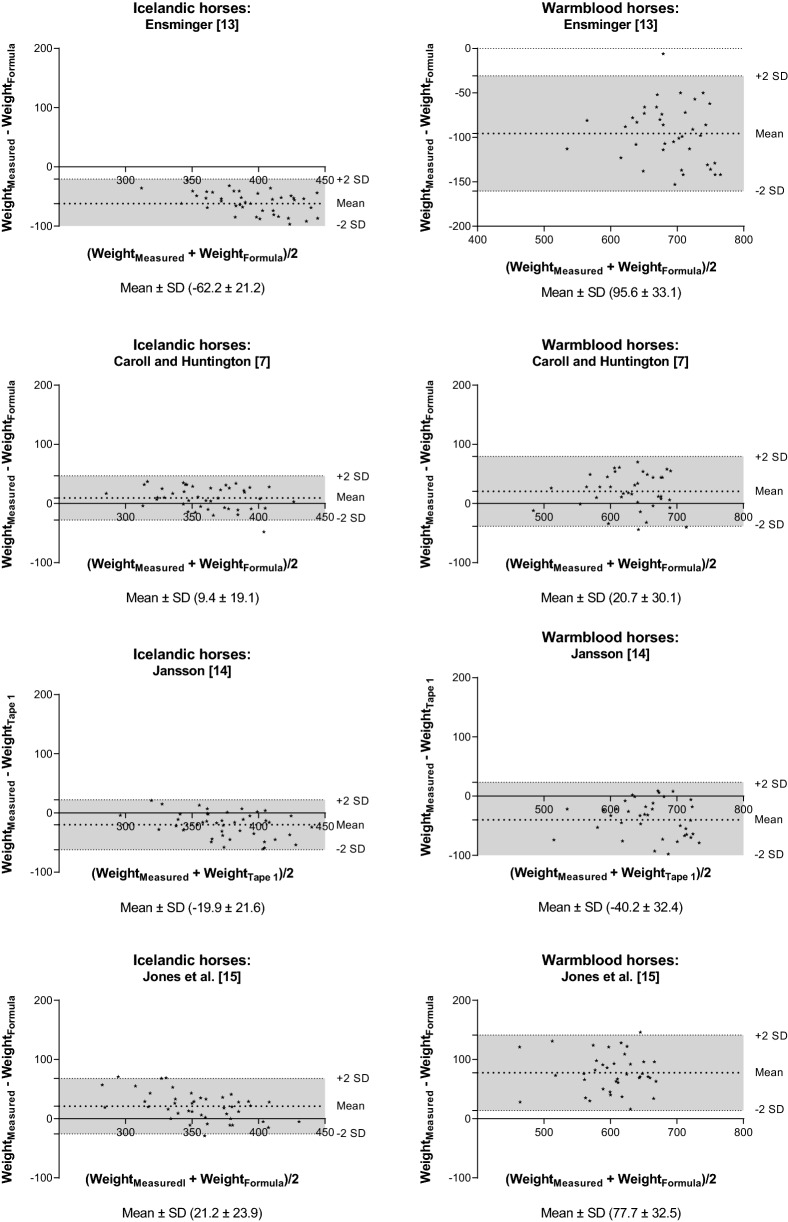
Bland–Altman plot of the measured and estimated body weight (BW). The BW of Icelandic and Warmblood horses was estimated based on length of the body and heart or umbilical girth. The y-axis is the difference between the measured and estimated BW (mean ± SD) and the x-axis is the average of the measured and estimated BW. The grey area represents the 95% confidence interval

**Fig. 4:**
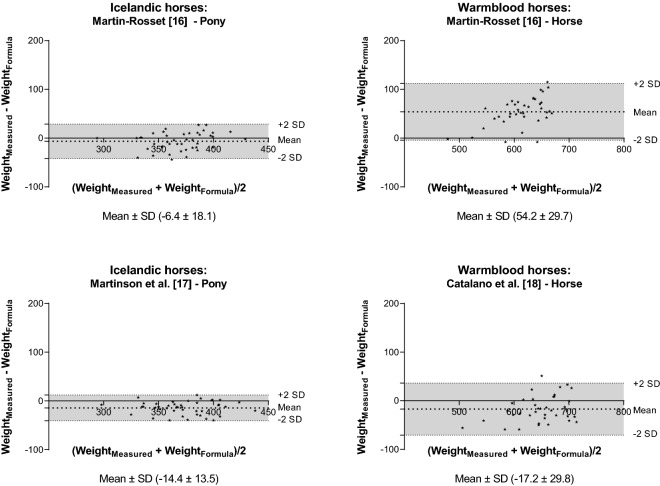
Bland–Altman plot of the measured and estimated body weight (BW). The BW of Icelandic and Warmblood horses was based on height at withers and heart girth alone or combined with length of the body and neck circumference. The y-axis is the difference between the measured and estimated BW (mean ± SD) and the x-axis is the average of the measured and estimated BW. The grey area represents the 95% confidence interval

Bland–Altman plots were used to assess the accuracy between measured and estimated BW, when BW was estimated based only on heart girth (Fig. 2), on length of the body and heart or umbilical girth (Fig. 3), or on height at withers and heart girth alone or combined with length of the body and neck circumference (Fig. 4). As it appears from Figs. 2, 3 and 4 the agreement between the measured and estimated BW varied largely depending on which BW formula that had been used for the estimation. The Bland–Altman plots and a combination of formula evaluation metrics (RMSPE, RSR, MB and SB) were used to assess formula performance, and all results are presented in Table 3.

**Table 3:** Results from the statistical analysis^d^ of the different formulas used to estimate body weight (BW) of Icelandic and Warmblood horses

References	Measurements^c^	Breed	RMSPE, kg	RSR	MB, %	SB, %	CCC	Mean ± SD, kg^e^
Marcenac and Aublet [10]	G2	Icelandic	25.4	0.87	32.4	19.5	0.71	14.4 ± 21.1
		Warmblood	37.2	0.71	4.6	17.1	0.76	8.1 ± 36.9
Staun [11]	G2	Icelandic	37.7	1.29	70.4	7.8	0.52	− 31.8 ± 20.7
		Warmblood	38.7	0.74	30.6	1.1	0.67	21.2 ± 32.7
Willoughby^a^ [12]	G2	Icelandic	20.5	0.70	0.6	35.0	0.80	1.7 ± 28.9
		Warmblood	42.0	0.80	23.9	19.8	0.74	− 20.6 ± 37.1
Ensminger [13]	G1 + L1	Icelandic	65.6	2.25	89.9	5.0	0.32	62.2 ± 21.2
		Warmblood	101.0	1.92	89.5	2.0	0.33	95.6 ± 33.1
Jansson [14]	G2 + L2	Icelandic	29.1	1.00	46.7	20.0	0.67	− 19.9 ± 21.6
		Warmblood	51.3	0.98	61.0	7.4	0.65	− 40.2 ± 32.4
Carroll and Huntington^b^ [7]	G2 + L1	Icelandic	21.1	0.72	20.1	21.5	0.78	9.4 ± 19.1
		Warmblood	36.2	0.69	32.7	6.1	0.78	20.7 ± 30.1
Jones et al. [15]	U + L1	Icelandic	31.8	1.09	44.6	28.2	0.66	21.2 ± 23.9
		Warmblood	84.0	1.60	85.4	1.6	0.39	77.7 ± 32.5
Martin-Rosset [16]	H + G2	Icelandic	18.9	0.65	11.5	1.1	0.76	− 6.4 ± 18.1
		Warmblood	61.7	1.17	77.3	1.2	0.46	54.2 ± 29.7
Martinson [17] Catalano [18]	H + NC + G1 + L1	Icelandic	19.6	0.67	53.9	2.7	0.80	14.4 ± 13.5
	H + NC + G1 + L1	Warmblood	32.0	0.61	28.9	0.1	0.79	− 17.2 ± 27.3

^a^In the original reference units are in inches (in) and pounds (lb).

^b^If BCS < 2.5 out of 5 use Y derivative 12,265, if BCS ≥ 3 out of 5 then use Y derivative 11,706

^c^Measurements are illustrated in Fig. 1

^d^Root mean square prediction error (RMSPE), RMSPE to standard deviation of the measured BW ratio (RSR), mean bias (MB) and slope bias (SB) deviations, concordance correlation coefficient (CCC), standard deviation (SD)

^e^Results from the Bland–Altman analysis [22]

In general, when the RMSPE and RSR were low the CCC was high, and the MB and SB deviations were used to identify why one formula performed better than another. There was also concordance between the MB and SB and the mean ± SD from the Bland–Altman analysis, respectively. One advantage of the Bland–Altman analysis is that the mean indicates whether BW is over- or underestimated.

Of the simple formulas only using heart girth circumference the formula from Marcenac and Aublet [10] and Willoughby [12] had higher CCC than the formula from Staun [11]. Of the formulas using two body measurements the formula from Carroll and Huntington [7] had a higher CCC than the rest followed by the formula from Martin-Rosset [16] for the Icelandic horses. The MB and SB showed that this was mainly explained by Carroll and Huntington [7] (and Martin-Rosset [16] for Icelandic horses) having relatively lower MB than the other formulas. Finally, the more complex formulas with four body measurements had high CCC (Martinson [17] and Catalano [18]), and they also had slightly lower RMSPE than the best formulas using single or two body measurements.

The plasma hormone concentrations for each breed are presented in Table 4. Leptin and insulin were both higher (P < 0.001) in the Icelandic than in the Warmblood horses.

**Table 4:** Plasma concentrations of leptin, insulin and cortisol in Icelandic and Warmblood horses

	Warmblood horses (n = 38)	Icelandic horses (n = 35)	P-value
Leptin (ng/mL)	0.350 ± 0.037	0.657 ± 0.039	< 0.001
Insulin (µU/mL)	7.16 ± 1.39	14.33 ± 1.45	< 0.001
Cortisol (ng/mL)	39.96 ± 2.11	38.94 ± 2.19	0.74

Values are presented as least-square means ± standard error of the mean

The associations between the variables CNS, BCS (1–5), BCS (1–9), leptin, insulin and cortisol are presented in Table 5. There were positive correlations between leptin and CNS, BCS (1–5), BCS (1–9) and insulin, and between insulin and CNS and BCS (1–5). The CNS, BCS (1–5) and BCS (1–9) are all strongly correlated.

**Table 5:** Spearman’s rank correlation coefficient (r_s_) between the measured variables^a^ in Icelandic and Warmblood horses (n = 73)

Correlation variables	r_s_	P-value
Leptin × CNS	0.37	< 0.01
Leptin × BCS (1–5)	0.38	0.01
Leptin × BCS (1–9)	0.34	0.01
Insulin × CNS	0.32	< 0.01
Insulin × BCS (1–5)	0.30	0.01
Insulin × BCS (1–9)	0.10	0.42
Cortisol × CNS	0.05	0.69
Cortisol × BCS (1–5)	0.05	0.70
Cortisol × BCS (1–9)	− 0.09	0.44
Leptin × insulin	0.60	< 0.001
Leptin × cortisol	0.04	0.71
Insulin × cortisol	0.12	0.30
CNS × BCS (1–5)	0.70	< 0.001
CNS × BCS (1–9)	0.48	< 0.001
BCS (1–5) × BCS (1–9)	0.58	< 0.001

^a^Measured variables: CNS: cresty neck score; BCS (0–5): body condition score on scale 0–5; BCS (1–9): body condition score on scale 1–9; BW, leptin, insulin and cortisol.

## Discussion

Knowledge of a horse’s accurate BW as well as BCS and CNS is essential in monitoring a horse’s nutritional plan and health. The main objective of this field study was to evaluate which of many developed BW formulas that accurately determines the BW of two different horse breeds, the Icelandic and Warmblood horse. Furthermore, it was the intention to assess the associations between the variables CNS, BCS, plasma concentrations of leptin, insulin and cortisol.

The formulas used in this study ranged from very simple ones based on a single measurement to such that were more complex, based on two to four different measurements. Based on the Bland–Altman analysis and the formula evaluation metrics there were formulas for which the deviation from the measured BW was relatively large and those formulas cannot be recommended to be used for the two breeds investigated here. It can be challenging to compare different formulas, and the trade-off between model complexity and predictive ability should be considered, i.e. a simple formula might not be as precise as a more complex one, but it might be easier to use in practice. Furthermore, based on the analysis performed in this study, the MB and SB deviations as well as the Bland–Altman analysis can be used to identify the strength and weakness of a formula. In general, the MB was larger than the SB deviation and this was also reflected in the Bland–Altman analysis were only few formulas had a mean close to zero (indicating whether and to what extent the BW is over- or underestimated).

Out of the formulas only using heart girth, the largest CCC was found when using the formulas from Marcenac and Aublet [10] and Willoughby [12]. Furthermore, the formula from Marcenac and Aublet [10] resulted in a mean value closer to zero based on the Bland–Altman analysis for both breeds than the one by Staun [11] which either over- (Warmblood) or underestimated (Icelandic) BW. Therefore, the results of this study suggest that the formulas from Marcenac and Aublet [10] and Willoughby [12] give the most accurate estimates of BW for both Icelandic and Warmblood horses when only heart girth is measured. For the Icelandic horses the CCC was largest for the formula from Willoughby [12] as compared to that of Marcenac and Aublet [10], whereas the opposite applied for the Warmblood horses.

There are several weight tapes available on the market which estimate BW based on heart girth only. Hoffman et al. [9] found no difference between BW estimated with a weight tape and measured BW in a small group of Icelandic horses (n = 13). In another study on Icelandic horses (n = 254) with different BCS, Jensen et al. [4] found that two weight tapes of different brand gave different results (BW was not measured), but with increasing BCS both heart girth and estimated BW increased. This highlights the strength and weaknesses of BW formulas. A weakness is that BWF only provide an estimate of the BW and the result will differ depending on which BWF that is used. However, a strength is that BWF (and weight tapes) can be an appropriate tool to estimate differences in BW among individual animals, or changes in BW over time in a single individual animal, if no weight scale is available. Ellis and Holland [5, 24] did also find different estimates of BW when using different brands of weight tapes and they suggested that height specific weight tapes should be used. Reavell [25] found that including length of the body when estimating BW in a group of horses of mixed breeds (n = 30) would improve the accuracy of the estimated BW. Hence, including other measurements than heart girth seems logic because of the large variation in conformation both between breeds and within breeds.

Among formulas including length and heart or umbilical girth, the one suggested by Carroll and Huntington [7] (n = 372) gave the best estimate of BW for both Icelandic and Warmblood horses, (the CCC was relatively higher for this formula than the others). It was also clear that the formula from Ensminger [13] had a low CCC and a high MB for the two breeds, and this was also present for the one from Jones et al. [15] for Warmblood horses. Carroll and Huntington [7] fitted the denominator, also known as the “Y” derivative, in their formula to obtain an accurate BW. Other authors have suggested the use of alternative “Y” derivatives to be more accurate for estimating BW depending on their study population [9, 26]. However, it is likely that an alternative “Y” derivative in most cases will improve the estimate of BW depending on e.g. sample size and type of equines used in the study. Therefore, the formula by Carroll and Huntington [7] is to recommend for both breeds if both length and heart girth is measured.

Further, more morphometric measurements such as height and neck circumference have been included in BW formulas in an attempt to enhance the accuracy of BW estimations [17, 18]. The formula including height and heart girth from Martin-Rosset [16] was accurate for Icelandic horses, but the opposite was found for Warmblood horses, where the MB was high. The complex formulas suggested by Martinson et al. [17] (n = 53) and Catalano et al. [18] (n = 89) were both accurate for estimating BW of Icelandic and Warmblood horses, respectively, in this study. However, their complexity might be a challenge when the formulas are applied to practise, because more measurements are required.

A challenge with BW formulas is the large number of horse breeds and the differences in conformation between them that makes it difficult to develop a single formula suited for all horses. Icelandic horses are purebred whereas Warmblood horses rather represent a type of horse as they originate from a mix of horses from different breeding associations with open studbooks. Therefore, large differences in conformation (e.g. height, length of body, light or heavy build) that might affect BW occur among Warmblood horses. The average BCS and CNS will affect the estimate of BW and these measurements had higher values for the Icelandic horses than the Warmblood horses in this study suggesting a higher body fat content. A large number of horses covering the entire range of BCS and CNS would be required if breed specific formulas should be developed, and therefore no new formulas are suggested based on the results of this study. However, this study showed that formulas developed for other breeds can be used for Icelandic horses, and the accuracy of the formulas were at the same level as for the Warmblood horses in this study. The simple formulas from Willoughby [12] and Carroll and Huntington [7] as well as the more complex formula from Catalano et al. [18] performed better (higher CCC) than the other formulas for Icelandic horses. Interestingly, there was clear differences in the MB and SB deviations between the three formulas. As complexity increases it might be more difficult to apply formulas to practice, e.g. measuring body length accurately might be difficult if a person is alone and more measurements require more sophisticated calculations.

Different subjective methods have been used to evaluate body fat accumulation in horses and ponies, and the most commonly used is the 9-point Henneke BCS system originally developed for use in Quarter horse broodmares, where BCS is categorized on a scale from 1 (poor) to 9 (extremely fat) [2]. The two BCS systems used in this study were strongly correlated, indicating that both can be used to describe BCS of Icelandic and Warmblood horses. A 5-point BCS system is commonly used in Iceland [27], however, the original paper is only available in Icelandic language, thus limiting its use. A translation of the system and a comparison to other systems would help for standardizing BCS of Icelandic horses.

There was only an effect of breed, not BCS, on insulin and leptin concentrations. An explanation might be that more variation in BCS is needed than that found in the present study population. The differences in BCS and CNS for Icelandic and Warmblood horses might still explain the differences in plasma concentrations of leptin and insulin between the two breeds, as leptin is an adipose tissue derived hormone related to the regulation of energy balance and it has been found that plasma leptin increases with increasing BCS in horses [20, 28] as found in this study. Ragnarsson and Jansson [29] did also find higher plasma concentrations of insulin in Icelandic horses with a BCS of ~ 7.4 (on the 9-points scale) than in Standardbred horses with a BCS of ~ 4.5, and they suggested that this relationship was due to differences in BCS more than breed differences. Furthermore, it has been found that leptin and insulin are correlated [30] in accordance with the results presented here. Since there were differences in BCS between the two breeds, it could have been interesting to test the horses for insulin regulation, as obesity and insulin dysregulation are important risk factors related to equine metabolic syndrome and endocrinopathic laminitis in horses [31]. This study has highlighted some simple tools for monitoring BW, an important measure when monitoring the nutritional status of horses for minimizing the risk of diseases.

## Conclusions

Body weight formulas only give an estimate of body weight and not a completely correct determination. In conclusion, this study showed that some simple and more complex formulas can be used for Icelandic horses even though they are not developed for this breed. Complex formulas using four morphometric measurements were accurate, but simple formulas using only heart girth, or heart girth and length can be used to estimate body weight and thereby be applied to weight tapes and used to estimate the body weight of both Icelandic and Warmblood horses. Plasma concentrations of leptin and insulin were higher for the Icelandic than the Warmblood horses, probably reflecting higher body fat content as suggested by the differences in BCS.

## Data Availability

The datasets used and/or analyzed during the current study are available from the corresponding author on reasonable request.

## Abbreviations

BW: body weight
BCS (0–5): body condition score on scale 0–5
BCS (1–9): body condition score on scale 1–9
CCC: concordance correlation coefficient
CNS: cresty neck score
G1: heart girth at withers
G2: heart girth at slope
H: height at withers
L1: length from point of shoulder to point of hip
L2: length from point of elbow to point of hip
Max: maximum
MB: mean bias
Min: minimum
NC: neck circumference
RMSPE: root mean square prediction error
RSR: RMSPE to standard deviation of the measured BW ratio
SB: slope bias
SD: standard deviation
U: umbilical girth
